# Influence of Gender on Cardiac and Encephalic Inflammation in the Elderly with Cysticercosis: A Case Control Study

**DOI:** 10.1155/2012/540858

**Published:** 2012-09-26

**Authors:** Camila Lourencini Cavellani, Rosana Rosa Miranda Corrêa, Mara Lúcia Fonseca Ferraz, Laura Penna Rocha, Ana Carolina Guimarães Faleiros, Ruy de Souza Lino Junior, Marlene Antônia dos Reis, Vicente de Paula Antunes Teixeira

**Affiliations:** ^1^Biological Sciences Department, General Pathology Discipline, Triângulo Mineiro Federal University, Disciplina de Patologia Geral, Rua Frei Paulino no. 30, Bairro Abadia CEP: 38025-180 Uberaba, MG, Brazil; ^2^Biological Sciences Department, Cellular Biology Discipline, Triangulo Mineiro Federal University, Uberaba, Minas Gerais, Brazil; ^3^General Pathology Sector, Institute of Tropical Pathology and Public Health, Goiás Federal University, Goiânia, Goiás, Brazil

## Abstract

*Background*. The present study explores the influence of the host's age and gender upon the inflammatory infiltrate. We aimed to quantify the inflammatory infiltrate caused by cysticercosis, which is related to aging, in the heart and in the encephalon. *Methods*. 75 autopsy protocols with cysticercosis diagnosis from department of pathology at a university hospital from 1970 to 2008 were reviewed. Two groups were formed: elderly with cysticercosis and nonelderly with cysticercosis. We used KS-300 (Kontron-Zeiss) software for morphometric analysis of the inflammation. *Results.* The elderly had an average of 3.1 ± 2.5 cysticerci, whereas the non-elderly had 2.7 ± 3.8 parasites. The non-elderly group with cysticercosis had significantly more inflammation, both cardiac and encephalic, than the elderly group. The elderly females with cysticercosis had more cardiac and encephalic inflammation. *Conclusions*. In this study, we showed that the non-elderly had significantly more cardiac and encephalic inflammation than the elderly, and that such inflammatory infiltrate decreases with age and depends upon the evolutionary stage of the cysticercus. Furthermore, there are differences concerning gender in the intensity of the inflammatory response due to cysticerci in the heart and brain parenchyma during senescence. Even during this period, women continue to have a more intense response to the parasitosis.

## 1. Introduction

Understanding the changes occurring within an aging immune system is essential if public health authorities are to be equipped to deal with an aging population. Specifically, knowledge of altered immune responses to infectious agents is required if rational clinical interventions are to be tailored to these aging individuals [1].

Aging is a continuous and slow process that compromises the normal functioning of various organs and systems [2]. As the population ages, there is growing interest in understanding host-parasite interaction and eventual prevention of chronic parasitic diseases, including cysticercosis, in elderly individuals.

Cysticercosis is emerging as a serious public health problem in many poor countries in Latin America, Africa and Asia. Although theoretically easy to control, and declared eradicable, cysticercosis remains neglected in most endemic countries [3]. This parasitosis may be asymptomatic or it may cause a variety of clinical manifestations depending on the number, location, and stage of cysticercus lesions. Pleomorphic disease is a result of the presence of the parasite itself (cysticerci), of the inflammatory process that surrounds the larvae, and of residual fibrosis and calcification [4–6]. It is likely that the combination of several factors is responsible for such differences, one of which may be gender-related [7].

The relevance of gender in host susceptibility has been explored in cysticercosis infection. In experimental murine *Taenia crassiceps* cysticercosis, female mice were found to be more susceptible than males in different syngeneic and congenic strains of mice [8]. The finding that gonadectomy equalized susceptibility between sexes, by reducing parasite loads in females and increasing it in males, first clearly indicated the relevance of sexual hormones [9].

The cysticercus contains a large number of antigens that can elicit a host immune inflammatory reaction. The inflammatory cellular infiltrate, if present, may be discrete with lymphocytes and eosinophils in the initial stage, or it may be a more intense lymphocyte infiltrate with giant multinucleated foamy macrophages in the necrotic stage [10].

Recent evidence suggests that immunosenescence associated to an immunological alteration caused by cysticercosis leads to a favorable condition for neoplasia development in elderly individuals attacked by the parasitosis. Moreover, it is likely that the patients continue to be infected with cysticercosis as they age [11].

The aim of this study was to quantify the inflammatory infiltrate in the heart and in the encephalon of the elderly with cysticercosis. Our hypothesis is that it is possible to quantify the infiltrated inflammatory among male and female elderly patients, due to the fact that those individuals are undergoing immunosenescence.

## 2. Material and Methods

### 2.1. Ethical Aspect

This research paper was approved by Triangulo Mineiro Federal University Research Ethics Committee under protocol no. 486. As this research regards autopsy material, the only risk was the loss of confidentiality. However, as a precautionary measure, the cases were identified by letters and numbers. Also, consent for the autopsy was given in writing by the next of kin after the death of the patient. Then the document was filed in the general hospital and the general pathology discipline records.

A retrospective transversal study of 3639 autopsies of adults collected at the General Hospital of Triangulo Mineiro Federal University, located in Uberaba, MG, Brazil, from 1970 to 2008, was carried out. Diagnosis of cysticercosis was made through histological demonstration or through direct visualization of the cysticercus, meeting the diagnostic criteria proposed by other authors [12], in 75 autopsies, 55 non-elderly, and 20 elderly patients. None of the patients included in the study were diagnosed with cysticercosis before autopsy. Information regarding age, gender, body weight, height, heart weight, brain weight, and the number, location, and evolutionary stage of the cysticercus was registered.

### 2.2. Material Preparation

In order to analyze the heart and brain inflammatory infiltration, we obtained 33 (13 elderly and 20 non-elderly) samples of heart and brain of patients with cysticercosis the 7 heart samples, amongst which 3 belonged to elderly patients and 4 belonged to non-elderly patients with cardiac cysticercosis, and 26 brains with neurocysticercosis, 10 of which belonged to elderly patients and 16 to non-elderly patients. The other organs were not found at the anatomical specimens' archives from the department of general pathology. The brain and heart samples affected by cysticercosis were fixed in formaldehyde 10% and subjected to routine histological processing so as to obtain 4 *μ*m thick sections, stained with hematoxylin and eosin (HE), for general morphological analysis and for quantification of the inflammatory cell infiltrate.

### 2.3. Morphometry

A video camera coupled to a standard light microscope and an interactive image analysis system (KS 300 Carl Zeiss) were used. We analyzed ten fields per quadrant; that is, 40 measurements were carried out in each slide. The representative number of measurements was determined through the method of Accumulated Means [13].

### 2.4. Inflammatory Cell Infiltrate

The HE-stained slides were examined using a standard light microscope with a 20x objective and 800x magnification range. The digital image showed the field where the number of inflammatory cells was counted in absolute value. Quantification was carried out by the observer's identification of such cells and through the staining performed by point-counting method.

### 2.5. Statistical Analysis

The variables were tested in order to verify the type of distribution using the Kolmogorov-Smirnov test and variance analysis. Student's *t*-test (*t*) or Mann-Whitney (*T*) was used in the comparison of two groups, and ANOVA (*F*) or Kruskal-Wallis (*H*) for comparison between three or more groups, followed by Bonferroni or Dunn test when necessary. Correlations between two variables were analyzed by Pearson's or Spearman correlation coefficient (*r*). Differences in significance levels of less than 5% (*P* < 0.05) were considered statistically significant. 

## 3. Results

Amongst the patients with cysticercosis, the average age of the non-elderly was 47.3 years, ranging from 23 to 58 years old, whereas the elderly had an average age of 66.7 years, ranging from 61 to 75 years old. Male and Caucasian patients predominated in both groups, and analysis of nutritional status showed that the non-elderly had an average body mass index (BMI) of 21.2 ± 4.4 kg/m^2^ and that the elderly with cysticercosis had an average BMI of 20.2 ± 9.9 kg/m^2^.

Heart weight and brain weight of the non-elderly were found to be higher than those of the elderly with cysticercosis, and both elderly and non-elderly male patients had heart weight and brain weight higher than female patients (Table 1).

It was possible to ascertain the evolutionary stage of the parasite in 8 cysticerci of elderly individuals, among whom 4 (50%) were Vesicular Stage, 2 (25%) Colloidal Vesicular Stage, 1 (12.5%) Granular Nodular Stage and (12.5%) Nodular Calcified Stage. Amongst the non-elderly, 4 (21.1%) cysticerci were in the first evolutionary stage, 5 (26.3%) Colloidal Vesicular Stage, 4 (21.1%) Granular Nodular Stage, and 6 (31.5%) Nodular Calcified Stage. The elderly had an average of 3.1 ± 2.5 cysticerci, whereas the non-elderly had 2.7 ± 3.8 parasites.

At all stages was observed some degree of inflammatory reaction around the cysticercus, its intensity decreased with the succession of evolutionary stages. The Colloidal Vesicular Stage showed the highest inflammatory infiltrate, followed by the Granular Nodular Stage in elderly and non-elderly groups (Table 2).

**Figure 1:**
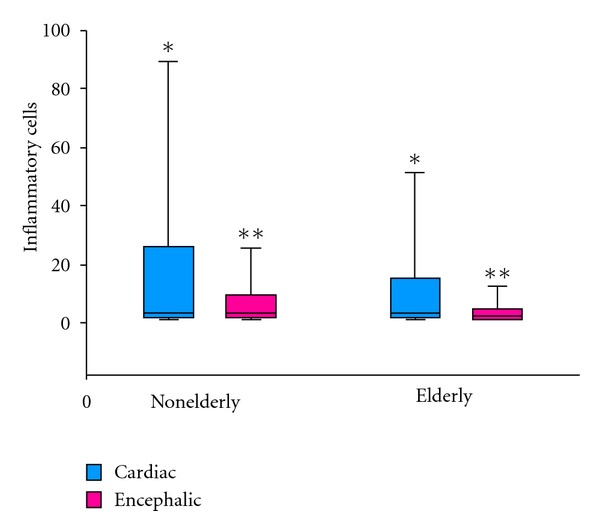
Comparison of the inflammatory infiltrate in heart and brain of non-elderly and elderly patients with cysticercosis.

**Figure 2:**
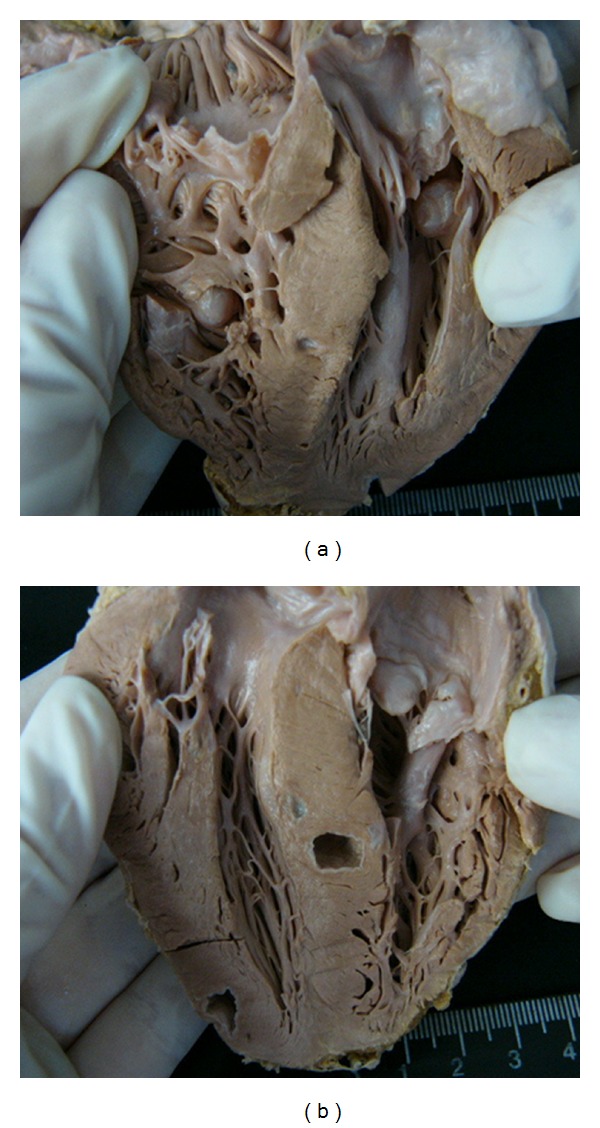
Cardiac cysticercosis in elderly individual. (a) Anterior view of the posterior half of organ showing two cysticerci in the endocardium. (b) Posterior view of the anterior half of organ showing cysticercus in the interventricular septum and in myocardium.

Analysis of the cardiac inflammatory infiltrate indicated that the non-elderly had significantly more inflammation than the elderly patients with cardiac cysticercosis (Figures 1 and 2).

In the non-elderly group, although men had more cardiac inflammation than women, this difference was not significant. Nonetheless, elderly females had significantly more inflammation than the elderly males (Table 3).

Encephalic inflammation was more acute amongst the non-elderly when compared to the elderly with neurocysticercosis (Figure 1). In the elderly group, female patients had significantly more encephalic inflammation than male patients. When contrasting both genders of the non-elderly group, male patients had more inflammation, yet without any significant difference (Table 3).

A positive and not significant correlation between encephalic inflammation and cardiac inflammation was found (*r* = 0, 032; *P* = 0,247), as well as a negative correlation between age and encephalic inflammation (*r* = −0,518; *P* = 0,03) or cardiac inflammation (*r* = −0,385; *P* = 0,186) in male group. Hence, it was demonstrated that the inflammation decreases with age in men. This relationship was not observed in female group.

## 4. Discussion

Population aging, which has increased since the last decades of the twentieth century, has changed the demographic and epidemiological profiles of countries such as Brazil. The increase in chronic degenerative diseases, which have replaced infectious and parasitic diseases, has demanded that more emphasis be placed on the prevention and treatment of such diseases, which leads to the need to know about their pathological changes during the aging process.

In the present study, heart and brain weights of non-elderly patients with cysticercosis were found to be higher than those of the elderly group with the parasitosis, without significant difference. Male patients had higher heart weight and brain weight, regardless of age. According to the literature, heart weight ranges from 347 g to 487 g in individuals over 60 years old, and brain weight ranges from 1105 g to 1264 g [14–16]. In an experimental study, not only did adult male rats have higher heart weight, but they also had larger myocardiocytes compared with female rats, which might be related to a higher risk of cardiovascular disease in males [17]. Studies involving patients without encephalopathy showed that the brain weight and the volume and density of the cell undergo a steady decrease with age, whereby male patients have higher brain weight than female patients [18].

Analysis of the inflammatory infiltrate showed that the non-elderly had significantly more cardiac and encephalic inflammation than the elderly, and that such inflammatory infiltrate decreases with age and depends upon the evolutionary stage of the cysticercus. The inflammatory process caused by cysticerci in the cerebral parenchyma and in the myocardium comprises mononuclear and polymorphonuclear cells, mainly eosinophils, macrophages, and lymphocytes [19, 20]. During the aging process, changes in the expression of functionally important cell receptors, reduction in the population of polymorphonuclear cells, and reduction in the capability of producing antibodies are verified, and these factors may lead to immune dysfunction [21, 22]. Therefore, our data might be related to changes in the immune response, mainly in T cells, which were found in the elderly individuals [23].

The Vesicular Stage was more prevalent among the elderly and Nodular Calcified Stage among non-elderly, and Colloidal Vesicular Stage showed higher inflammatory infiltrate in both groups. Researches show that a more intense inflammation with lymphocyte and macrophage infiltrate can be found around the cysticercus in Colloidal Vesicular Stage [10, 24, 25]. With cysts degenerates, the inflammatory reaction tends to decrease in the Granular Nodular Stage, denoting continuity in the host reaction against the parasite remnants without, however, having an association with the type or intensity of the inflammatory response [24, 26]. 

The duration of each of the progressive stages in the natural history of cysticercosis has not been established because there are considerable differences between individuals, particularly in relation to the intensity of the endogenous immune response [27]. Whereas the parasite typically dies few years after infection stimulating a vigorous inflammatory response, probably the acquisition of the parasite occurred most recently in the elderly than in non-elderly patients, or the elderly, due to changes in the immune system with aging, preserve the cysticercus in the initial phase for a long time. However, further research is needed.

Amongst the elderly with cysticercosis, the female patients had more occurrences of cardiac inflammation and encephalic inflammation. There are also some indications that, in human neurocysticercosis caused by *T. solium*, women show a more intense inflammatory profile in the cerebrospinal fluid than men do and, likewise, are more prone to develop a severe and generalized encephalitic process [28]. Women had evidence of cardiac and encephalic inflammation more frequently than men. These observations are in accordance with previous studies in which gender has been associated with the intensity of the inflammatory response against the parasite, possibly promoted by the female sex-steroid levels [7, 9, 28–32]. Therefore, our data showed that even during senescence, when a decrease in the levels of female sex steroids is noticed, women have a more intense immune response towards cysticercosis in comparison with men.

Cardiac and encephalic inflammation showed a positive correlation in both groups. Studies have showed that the presence of multiple parasites is more common in older individuals [28]. Encephalic inflammation and cardiac inflammation were more commonly found in the analyzed material, and most of the individuals had cysticercosis in more than one location.

It was found that multiple cysticerci lesions and multiple vesicular cysts were more frequently observed in the elderly without an increase in severity of the clinical symptoms. This observation could indicate that susceptibility to become infected increases with age, whereas susceptibility to follow a pathogenic course of the infection decreases. This suggestion finds additional support in the reduction of leukocyte counts with age. The reverse effect of age upon susceptibility to infection and to resistance against severe disease has been found in other parasite infections such as schistosomiasis [33, 34] and it suggests that susceptibility and pathogenicity involve distinct physiological pathways that are independently regulated [28].

This study presents important findings on the influence of gender on cardiac and encephalic inflammation in the elderly with cysticercosis, although it has some limitations, such as small number of samples for analyses, particularly of the gender influence, loss of many biopsies and retrospective design. Future researches are needed to determine the mechanisms of the differences related to gender and immunosenescence associated to immunological alteration caused by cysticercosis.

## 5. Conclusions

In this study, we showed that the non-elderly had significantly more cardiac and encephalic inflammation than the elderly, and that such inflammatory infiltrate decreases with age and depends upon the evolutionary stage of the cysticercus. Furthermore, there are differences, concerning gender, in the intensity of the inflammatory response due to cysticerci in the heart and brain parenchyma during senescence. Even during this period, women continue to have a more intense response to the parasitosis.

## Figures and Tables

**Table 1:** Heart and brain weight according to gender of the elderly and non-elderly patients with cysticercosis.

Groups	Gender	Heart weight (g)	Brain weight (g)
		Mean ± SD	
Elderly		347.2 ± 121.9	1216.1 ± 158.2
Nonelderly		370.1 ± 102.8	1228.3 ± 131.3
		*t*, *P* > 0,05	*t*, *P* > 0,05
Elderly	Female	299.8 ± 65.7	1231.0 ± 204.9
	Male	360.0 ± 149.8	1261.1 ± 107.9
Non-elderly	Female	336.6 ± 107.0	1166.7 ± 84.2^1^
	Male	395.2 ± 103.6	1313,6 ± 155.3^2^
		*F*, *P* > 0.05	*F*, *P* < 0.05

SD: standard deviation. *t*: Student's *t*-test; *F*: Anova; 1 × 2 Bonferroni test, *P* < 0.05.

**Table 2:** Comparison of the inflammatory infiltrate in relation the evolutionary stage of the cysticerci in brain of non-elderly and elderly patients with cysticercosis.

Groups	Vesicular stage	Colloidal vesicular stage	Granular nodular stage	Nodular calcified stage
	Median (minimum–maximum)			
Elderly	1.0 (1.0–4.0)	3.0 (1.0–12.0)	2.0 (1.0–6.0)	1.0 (1.0–3.0)
Non-elderly	1.0 (1.0–6.0)	4.0 (1.0–18.0)	2.5 (1.0–8.0)	2.0 (1.0–5.0)
	*T*, *P* > 0.05	*T*, *P* < 0.05	*T*, *P* < 0.05	*T*, *P* > 0.05

*T*: Mann-Whitney test.

**Table 3:** Comparison of the inflammatory infiltrate in heart and brain in relation to the gender of non-elderly and elderly patients with cysticercosis.

Groups	Gender	Cardiac inflammation	Encephalic inflammation
		Med (minimum–maximum)	
Elderly	Female	3.0 (1.0–51.0)^1^	3.0 (1.0–12.0)^3^
	Male	2.0 (1.0–23.0)^2^	2.0 (1.0–6.0)^4,5^
Non-elderly	Female	3.0 (1.0–16.0)	3.0 (1.0–10.0)
	Male	3.0 (1.0–89.0)	3.0 (1.0–25.0)^6^
		*H*, *P* < 0.05	*H*, *P* < 0.05

1 × 2, 3 × 4, 5 × 6: Dunn test, *P* < 0.05.
